# Divergent gene expression of *PTGS1* and *PTGS2* along the disease course of chronic myeloid leukaemia

**DOI:** 10.3389/fonc.2025.1556956

**Published:** 2025-05-15

**Authors:** Denise K. Wosniaki, Bianca N. Kusma, Anelis M. Marin, Eduardo C. Munhoz, João S. de H. Farias, Eduarda B. Mendes, Mateus N. Aoki, Dalila L. Zanette

**Affiliations:** ^1^ Laboratory for Applied Science and Technology in Health, Carlos Chagas Institute, Oswaldo Cruz Foundation (Fiocruz), Curitiba, Brazil; ^2^ Erasto Gaertner Hospital, Curitiba, Brazil

**Keywords:** PTGS1 and PTGS2, inflammatory process, leukaemogenesis, chronic myeloid leukemia, prostaglandin

## Abstract

**Introduction:**

Chronic myeloid leukaemia (CML) is a chronic myeloproliferative disorder caused by the reciprocal translocation of chromosomes 9 and 22. This genetic abnormality leads to the uncontrolled proliferation of myeloid lineage cells, ultimately causing leukaemia. Inflammation plays a significant role in the progression of various cancers, including lungs, colorectal, breast, head and neck, and haematological malignancies. Prostaglandins, which are key mediators in these processes, are synthesised by cyclooxygenases. Consequently, these genes are critical targets for studying CML due to their involvement in leukemogenesis.

**Methods:**

We investigated the expression levels of *PTGS1* and *PTGS2* genes in peripheral blood samples from CML patients. Quantitative PCR (qPCR) and in vitro cell culture experiments were used to assess gene expression. Statistical analysis included the Kruskal–Wallis test and simple linear regression models. Patients were stratified according to disease status, *BCR::ABL1* levels, treatment-free remission (TFR), and use of imatinib. The impact of NSAID use on gene expression was also evaluated.

**Results:**

Increased expression of *PTGS1* and *PTGS2* was observed in patients with favourable prognosis. Conversely, lower expression levels were found in patients with uncontrolled disease (high *BCR::ABL1* levels) compared to healthy controls, those in TFR, and patients undergoing imatinib treatment. NSAID use did not significantly alter *PTGS* gene expression. In vitro treatment of a CML cell line with imatinib also showed increased *PTGS1* expression.

**Discussion:**

These findings suggest a potential role of inflammatory pathways in CML progression and treatment response. The upregulation of *PTGS1* and *PTGS2* in patients with controlled disease and after imatinib treatment may indicate a link between COX enzyme activity and leukemogenesis. Further investigation is warranted to clarify the mechanistic role of these genes in CML pathophysiology and therapy outcomes.

## 1 Introduction

Chronic myeloid leukaemia (CML) is a subtype of leukaemia characterized mainly by a reciprocal translocation of chromosomes 9 and 22, designated as (9; 22) (q34; q11). This genetic alteration results in the Philadelphia chromosome and leads to the fusion of the *ABL1* and *BCR* genes (Murine Abelson on chromosome 9 and Breakpoint cluster on chromosome 22) (1). This fusion of the *BCR* and *ABL1* genes produces a chimeric transcript that translates into the *BCR::ABL1* chimeric oncoprotein, which acts as a constitutively active tyrosine kinase. This oncoprotein stimulates pathways that promote leukaemogenesis and the uncontrolled proliferation of myeloid cells (2).

The chimeric *BCR::ABL1* protein influences cell growth and survival pathways, resulting in changes to apoptosis and the activation of transcription factors (3). Tyrosine kinase inhibitors (TKIs), such as imatinib, nilotinib, and dasatinib, are pharmacological agents with significant effectiveness in managing CML. These agents can suppress leukaemic proliferation and even lead to complete remission, restoring the normal proportions of blood cells (3). However, a major challenge is the resistance developed by some patients, which can compromise treatment and potentially lead to the blast phase of the disease (4).

The relationship between cancer and inflammation is well established, with evidence indicating that cancer can originate in inflammation sites. Additionally, the tumor environment can induce an inflammatory process by recruiting immune cells to the tumor site. Neoplastic cells can also modify inflammatory reactions independently of immune cell recruitment by overexpressing pro-inflammatory factors such as proteases, cytokines, and eicosanoids, including prostaglandins (5). COX-1 and COX-2 are the enzymes encoded by *PTGS1* and *PTGS2* genes, respectively, playing an important part in prostaglandin biosynthesis. While COX-1 is constitutively expressed, COX-2 expression is induced by cytokines, growth factors, and carcinogenic stimuli. Given the central role of *PTGS2* in inflammation and the association between inflammation and cancer, it is reasonable to expect that *PTGS2* would be overexpressed in cancer. However, its expression is highly variable among cancer types and even within the same type (6). This variability also appears to be present in haematological malignancies, as reviewed by Ramon and colleagues. In CML, there are only a limited number of reports, primarily based on *in vitro* data using the erythroleukemia K562 cell line (7). In this regard, this study aimed to evaluate *PTGS1* and *PTGS2* gene expression in clinical samples from CML patients.

## 2 Materials and methods

### 2.1 Samples

Peripheral blood samples from CML patients were collected at the Erasto Gaertner Hospital (Curitiba, Brazil) following informed consent and approval from the local Ethics Committee (CAAE 08809419.0.0000.0098 and 53207021.5.0000.0098). Two cohorts were selected for this project: a primary cohort with a low number of samples and a secondary cohort with a larger number of subgroups. The expression of *PTGS1* and *PTGS2* targets was evaluated quantitatively by RT-qPCR in these cohorts of CML patients, divided into groups considering the following variables: age, sex, type and duration of treatment, and laboratory data.

Samples for these cohorts (summarised in **Table 1**) were categorised into 6 groups, inside 3 categories based on *BCR::ABL1* expression: ‘uncontrolled’ for high expression, ‘controlled’ for low or absent expression, and ‘never detected’ for HCs. The uncontrolled group was further subdivided into three subgroups. The first included 17 patients with the T315I mutation, the second consisted of 30 patients exhibiting high levels of *BCR::ABL1* transcripts after treatment with imatinib and with no mutations detected, and the third comprised 21 patients at diagnosis without any treatment. The controlled group was subdivided into two subgroups: the first included 11 patients treated with imatinib for a minimum of six months, while the second consisted of 17 patients who demonstrated significant progression and started a treatment-free-remission (TFR) protocol. The HC group included 16 samples from blood donors.

**Table 1:** Description of the analytic groups in analytic cohort.

Name	Classification	N°. of patients
HC	Never detected *BCR::ABL1*	16
TFR	Controlled BCR::ABL1	17
Imatinib	Controlled BCR::ABL1	11
Diagnosis	Uncontrolled *BCR::ABL1*	21
*BCR::ABL1* high	Uncontrolled *BCR::ABL1*	30
T315I	Uncontrolled *BCR::ABL1*	17

HC/controls, healthy blood donors; TFR, patients who achieved a good and functional response to imatinib and have begun a treatment-free remission protocol; imatinib, patients undergoing treatment with imatinib; diagnosis, patients diagnosed with CML but not treated; *BCR::ABL1* high, patients with a percentage of *BCR::ABL1* transcript higher than 0.1%; T315I, CML patients with the T315I mutation detected.

### 2.2 Buffy coat RNA and DNA extraction

After separating the blood phases by centrifugation (400 g, 10 min, 4°C), the buffy coat was utilised for acid nucleic extraction, which was subdivided into two fractions. One fraction was designated for total RNA extraction using the QIAamp^®^ RNA Blood Mini Kit (Qiagen), while the other was used for DNA extraction with QIAamp^®^ DNA Blood Mini Kit (Qiagen) following the manufacturer’s instructions. The extracted RNA and DNA were quantified in NanoDrop™ One (Thermo Scientific) and stored at -80°C until further use. The quality of the samples was assessed based on 230/260 and 260/280 absorbance ratios measured in Nanodrop.

### 2.3 Quantification of *BCR::ABL1*

Total RNA extracted from the buffy coat was analysed by RT-qPCR following the protocol described by Marin et al. (2023) (8). Briefly, reverse transcription and PCR amplification were performed as one-step reactions to evaluate the expression ratio between the *ABL1* endogenous gene and *BCR::ABL1* target transcriptional gene. Quantifications were based on a commercial standard curve, with the final results presented as the ratio of *ABL1* to *BCR::ABL1*, adjusted using a correction factor and curve intersection values.

### 2.4 Detection of resistance mutations

Five mutations known to confer resistance to TKIs in CML patients were analyzed: rs121913459 (T315I), rs121913448 (E255K), rs121913461 (Y253H), rs121913449 (E255V), and rs121913452 (V359F). Specific Taqman™ probes for each single nucleotide polymorphism (SNP) were used in qPCR reactions with TaqPath™ PromAmp™ Master Mix (Thermo Fisher Scientific). The PCR reactions were performed on a QuantStudio 5™ real-time PCR platform (Thermo Fisher Scientific) using the genotyping settings. The PCRs were standardized following the appropriate master mix protocol, including a blank control (non-template control, NTC).

### 2.5 Complementary DNA synthesis and *PTGS* expression analysis

The total RNA was reverse transcribed using the High-Capacity cDNA Transcription Kit (ThermoFisher Scientific), following the manufacturer’s instructions. For the relative quantification of the targets *PTGS1* and *PTGS2*, as well as the housekeeping gene *ACTB*, specific Taqman probes were used (ThermoFisher Scientific). The TaqMan™ Universal PCR Master Mix (ThermoFisher Scientific) was employed, and the PCR reactions were performed in duplicates on a QuantStudio 5™ real-time PCR platform using default cycling conditions and a negative control (NTC).

### 2.6 Statistical analysis

Demographic data, specifically age, were analyzed by Kruskall-Wallis. The relative expression of the target genes was calculated using the beta-actin (*ACTB*) gene as a normalizer, following the 2^-ΔΔCT^ methodology (9), for it *ACTB* was used as a normalizer and the mean of the ΔC (normalized values) of the health control group was used as a calibrator, obtaining the ΔΔC for each sample. The results obtained for single-point analysis were analyzed in GraphPad Prism 9. Kruskall-Wallis with Dunn’s *post-hoc* test was employed to compare the three groups of samples, with a p-value of <0.05 considered significant.

### 2.7 Imatinib *in vitro* tests

*In vitro* tests were performed using the K562 chronic myeloid leukemia cell line model by seeding 1x10^4^ cells in 96-well plates, containing 100µL of RPMI 1640 culture medium (Gibco) with 1% antibiotic (Penicillin and streptomycin, Gibco) and supplemented with 10% fetal bovine serum (Thermo Scientific), and were incubated at 37°C with 5% CO^2^.

The tests were performed by first evaluating the IC50 (50% inhibitory concentration) of the compound imatinib mesylate (imatinib mesylate was kindly provided by Dr. Luiz Claúdio Ferreira Pimentel from Farmanguinhos (Institute of Technology in Pharmacology – Fiocruz Rio de Janeiro). A concentration curve was built, with 8 concentration points, ranging from 5µM to 0.03µM (50% regression at each point), the curve was evaluated in 4 separate and independent experiments, always with 6 replicates for each concentration. The experiments also had control wells, 1) cells without addition of any compounds but the culture media, 2) cells with the drug vehicle (DMSO) and 3) cells containing SDS as a death control. The 3 independent experiments were evaluated using the Alamar blue technique (Resazurin, Sigma) at a concentration of 0.0315ug/mL and fluorescences were read in a Synergy microplate reader (BioTek Company) at wavelengths 560nm and 600nm. The last independent experiment was the confirmation test using the CellTiter-Glo^®^ 2.0 Cell Viability Assay kit (Promega) following the manufacturer’s recommendations, which was used to further confirm the results with resazurin. The IC50 concentration assessment was based on the “Equation: Biphasic dose-response” model in the GraphPad Prism software guidelines (https://www.graphpad.com/guides/prism/latest/curve-fitting/reg_biphasic_dose_response.htm).

To evaluate the expression of *PTGS1* and *PTGS2* in K562 cell line, the cells were passaged as described above in medium containing the IC50 concentration of Imatinib (0.4µM) and the effects of this Imatinib exposure on *PTGS* genes expression was evaluated by the same methods described previously for clinical samples in the topic “Complementary DNA synthesis and *PTGS* expression analysis” but now using the RNA isolated with RNeasy Mini Kit (QIAGEN). The exposure was analyzed at 3 timepoints after the Imatinib treatment, 4h, 24h and 48h. As a negative control, cells without any Imatinib treatment were analyzed. The relative expression of the target genes was calculated using the beta-actin (*ACTB*) gene as a normalizer, following the 2^-ΔΔCT^ methodology (9) where in this case just the normalization happened, without calibration. The results were analyzed in GraphPad Prism 9, using Simple Linear Regression models.

## 3 Results

### 3.1 Epidemiological description

**Figure 1:**
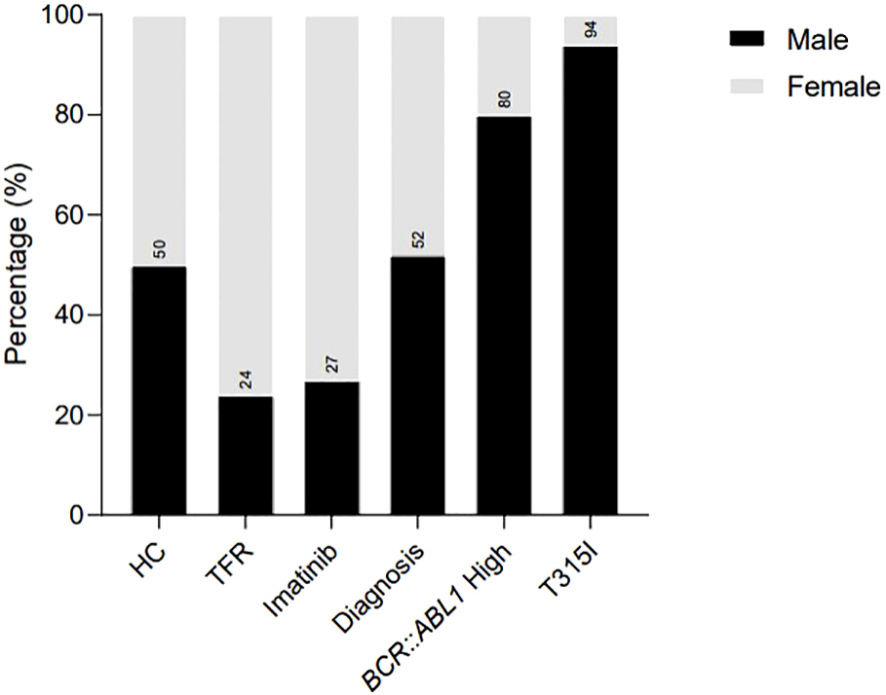
Sex distribution in the analytic cohort of patients recruited for the analysis, illustrating the percentage of females and males in each group. HC/controls, healthy blood donors; TFR, patients who achieved a good and functional response to imatinib and have begun a treatment-free remission protocol; imatinib, patients undergoing treatment with imatinib; diagnosis, patients diagnosed with CML but not treated; *BCR::ABL1* high, patients with a percentage of *BCR::ABL1* transcript higher than 0.1%; T315I, CML patients with the T315I mutation detected.

**Figure 2:**
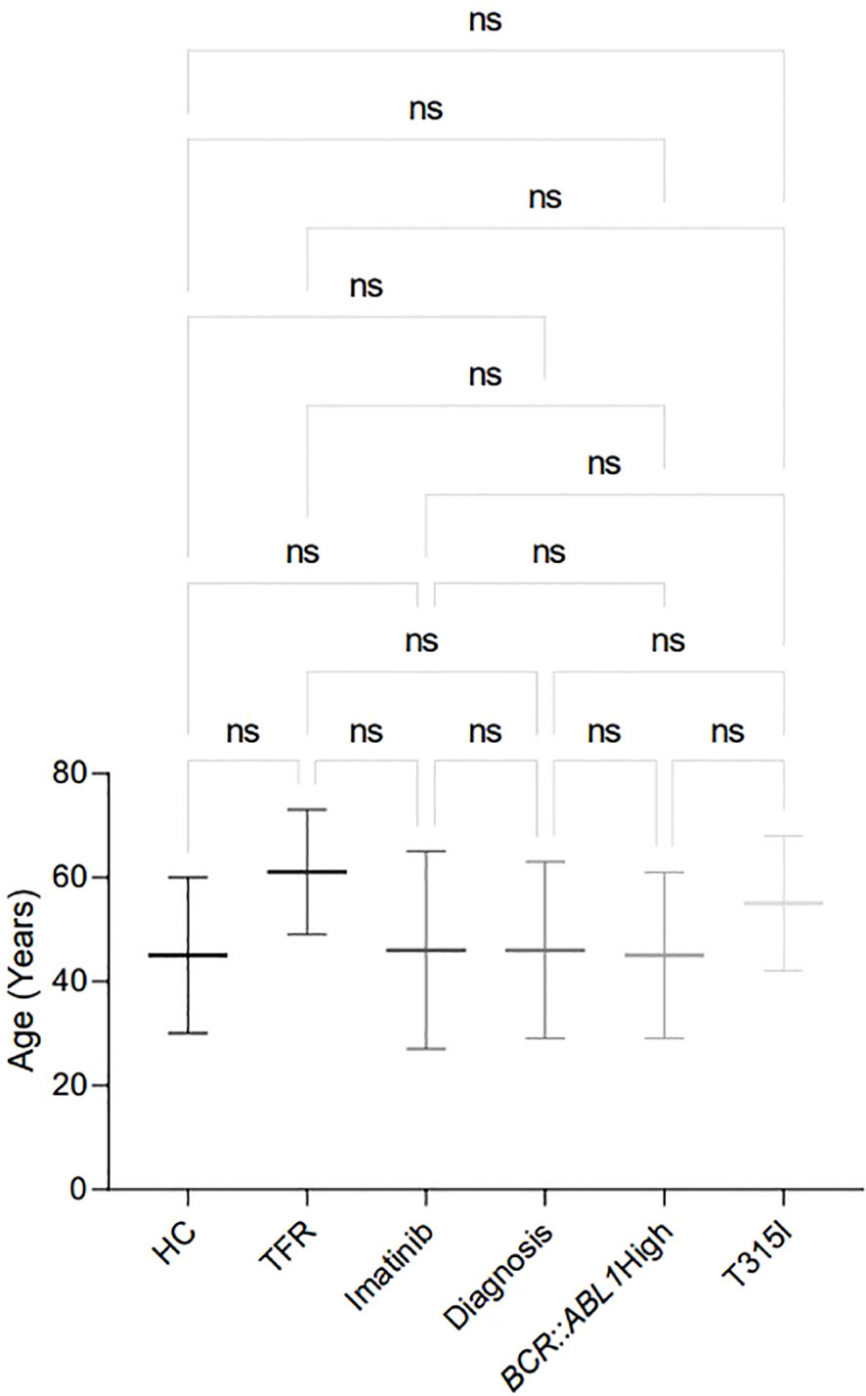
Age distribution in the analytic cohort of patients recruited for the analysis, showing the mean ± standard deviation of age for each analysed group. HC/controls, healthy blood donors; TFR, patients who achieved a good and functional response to imatinib and have begun a treatment-free remission protocol; imatinib, patients undergoing treatment with imatinib; diagnosis, patients diagnosed with CML but not treated; *BCR::ABL1* high, patients with a percentage of *BCR::ABL1* transcript higher than 0.1%; T315I, CML patients with the T315I mutation detected. ns: non-significant.

All patients selected for analysis were diagnosed with CML by medical professionals at the Erasto Gaertner Hospital and were at different stages of treatment. Six groups were established for analysis: the first group, T315I, comprised patients harboring this mutation, which confers resistance to treatment with imatinib. The second group consisted of samples from patients currently undergoing treatment with imatinib who still exhibited elevated expression of the *BCR::ABL1* transcript (>0.1%). The third group included patients at the diagnosis who had not received any treatment, while the fourth group comprised patients treated with imatinib for a minimum of six months. The fifth group was composed of patients who demonstrated significant progression in their treatment with imatinib and subsequently began a TFR protocol. The sixth group served as a control group, consisting of samples from healthy donors. **Figures 1** and **2**, along with **Table 2**, present the epidemiological data of the participants recruited for this analysis.

**Table 2:** P-values from the Kruskall–Wallis analysis showing the results of the multiple comparisons test related to age among the groups in the analytic cohort.

*PTGS1* groups	Multiple comparisons (p-value)
T315I vs *BCR::ABL1* high	>0.9999
T315I vs diagnosis	>0.9999
T315I vs imatinib	>0.9999
T315I vs TFR	>0.9999
T315I vs HC	>0.9999
*BCR::ABL1* high vs diagnosis	>0.9999
*BCR::ABL1* high vs imatinib	>0.9999
*BCR::ABL1* high vs TFR	>0.9999
*BCR::ABL1* high vs HC	>0.9999
Diagnosis vs imatinib	>0.9999
Diagnosis vs TFR	>0.9999
Diagnosis vs HC	>0.9999
Imatinib vs TFR	>0.9999
Imatinib vs HC	>0.9999
TFR vs HC	>0.9999

HC/controls, healthy blood donors; TFR, patients who achieved a good and functional response to imatinib and have begun a treatment-free remission protocol; imatinib, patients undergoing treatment with imatinib; diagnosis, patients diagnosed with CML but not treated; *BCR::ABL1* high, patients with a percentage of *BCR::ABL1* transcript higher than 0.1%; T315I, CML patients with the T315I mutation detected. Group 6 (HC/controls), healthy blood donors. A p-value of <0.05 was considered significant.

### 3.2 Gene expression analysis

**Figure 3:**
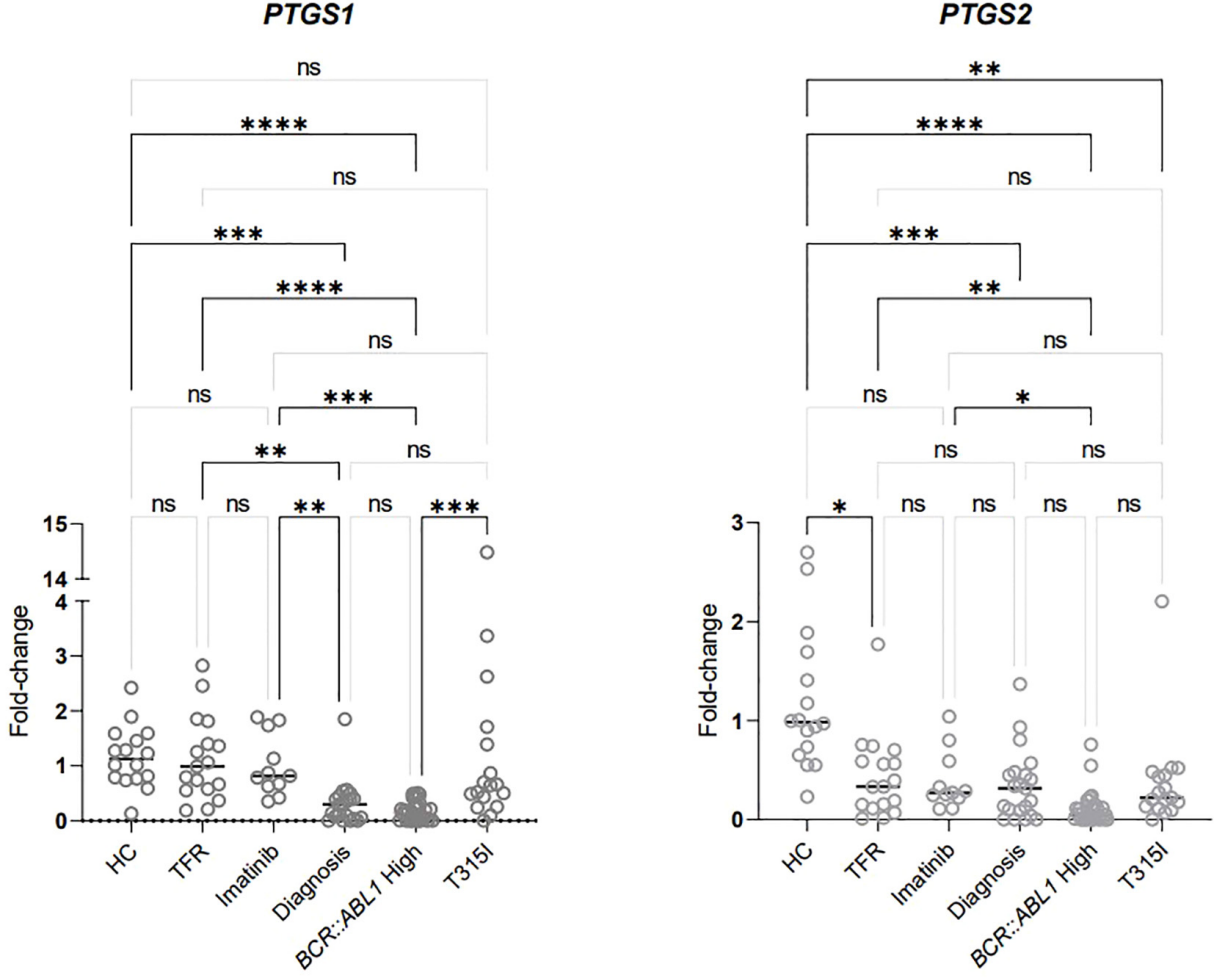
Analysis of *PTGS1* and *PTGS2* expression in RNA extracted from the buffy coat (data shown in **Table 4**). HC/controls, healthy blood donors; TFR, patients who achieved a good and functional response to imatinib and have begun a treatment-free remission protocol; imatinib, patients undergoing treatment with imatinib; diagnosis, patients diagnosed with CML but not treated; *BCR::ABL1* high, patients with a percentage of *BCR::ABL1* transcript higher than 0.1%; T315I, CML patients with the T315I mutation detected. A p-value of <0.05 was considered significant. *p < 0.05, **p < 0.01, ***p < 0.001, ****p < 0.0001. ns: non-significant.

The expression of the *PTGS1* gene was evaluated in RNA extracted from the buffy coats of patients using RT-qPCR and the 2^-ΔΔCT^ method to determine the fold changes between the groups. The analysis results are shown in **Tables 3** and **4** and **Figure 3**.

**Table 3:** P-values from the Kruskall–Wallis analysis showing the general rank p-value and the mean ± standard deviation of each group calculated using the 2^-ΔΔCT^ method.

Gene	General rank	Mean ± standard deviation					
		T315I	BCR::ABL1 High	Diagnosis	Imatinib	TFR	HC
*PTGS1*	**<0.0001**	3.8 ± 1.5	0.1 ± 0.1	0.3 ± 0.4	1.0 ± 0.5	1.1 ± 0.7	1.1 ± 0.5
*PTGS2*	**<0.0001**	0.3 ± 0.4	0.1 ± 0.1	0.3 ± 0.3	0.3 ± 0.2	0.4 ± 0.4	1.1 ± 0.7

HC/controls, healthy blood donors; TFR, patients who achieved a good and functional response to imatinib and have begun a treatment-free remission protocol; imatinib, patients undergoing treatment with imatinib; diagnosis, patients diagnosed with CML but not treated; *BCR::ABL1* high, patients with a percentage of *BCR::ABL1* transcript higher than 0.1%; T315I, CML patients with the T315I mutation detected. A p-value of <0.05 was considered significant.

Bold values indicate statistically significant differences.

**Table 4:** P-values of the Kruskall–Wallis analysis showing the results of the multiple comparisons test.

Analytic groups	*PTGS1*	*PTGS2*
**T315I vs *BCR::ABL1* high**	**0.0031**	0.0976
T315I vs diagnosis	0.1217	>0.9999
T315I vs imatinib	>0.9999	>0.9999
T315I vs TFR	>0.9999	>0.9999
T315I vs HC	>0.9999	**0.0090**
*BCR::ABL1* high vs diagnosis	>0.9999	0.1796
***BCR::ABL1* high vs imatinib**	**0.0001**	**0.0311**
***BCR::ABL1* high vs TFR**	**<0.0001**	**0.0101**
***BCR::ABL1* high vs HC**	**<0.0001**	**<0.0001**
**Diagnosis vs imatinib**	**0.0063**	>0.9999
**Diagnosis vs TFR**	**0.0011**	>0.9999
**Diagnosis vs HC**	**0.0001**	**0.0008**
Imatinib vs TFR	>0.9999	>0.9999
Imatinib vs HC	>0.9999	0.1982
TFR vs HC	>0.9999	**0.0487**

HC/controls, healthy blood donors; TFR, patients who achieved a good and functional response to imatinib and have begun a treatment-free remission protocol; imatinib, patients undergoing treatment with imatinib; diagnosis, patients diagnosed with CML but not treated; *BCR::ABL1* high, patients with a percentage of *BCR::ABL1* transcript higher than 0.1%; T315I, CML patients with the T315I mutation detected. A p-value of <0.05 was considered significant.

Bold values indicate statistically significant differences.

*PTGS1* expression was significantly upregulated in HCs compared to patients undergoing treatment with high *BCR::ABL1* levels and those diagnosed without treatment. Additionally, *PTGS1* levels were elevated in the two groups of patients classified as having good progression (imatinib and TFR) compared to those categorized as having bad progression (*BCR::ABL1* high and diagnosis). The expression of this gene was not significantly different between the groups with good progression. Furthermore, it was significantly higher in patients with the T315I mutation compared to those with elevated *BCR::ABL1* levels.

Significantly decreased expression of *PTGS2* was observed in patients with poor progression (T315I, *BCR::ABL1* and diagnosis) compared to HCs. The *BCR::ABL1* high group showed significant downregulation of *PTGS2* compared to all three groups classified as having good progression (imatinib, TFR, and HC). In contrast, the T315I and diagnosis groups exhibited similar downregulation as verified in the *BCR::ABL1* high group. The TFR group was also downregulated compared to HCs, although it is noteworthy that the p-value for this comparison was 0.0487.

**Figure 4:**
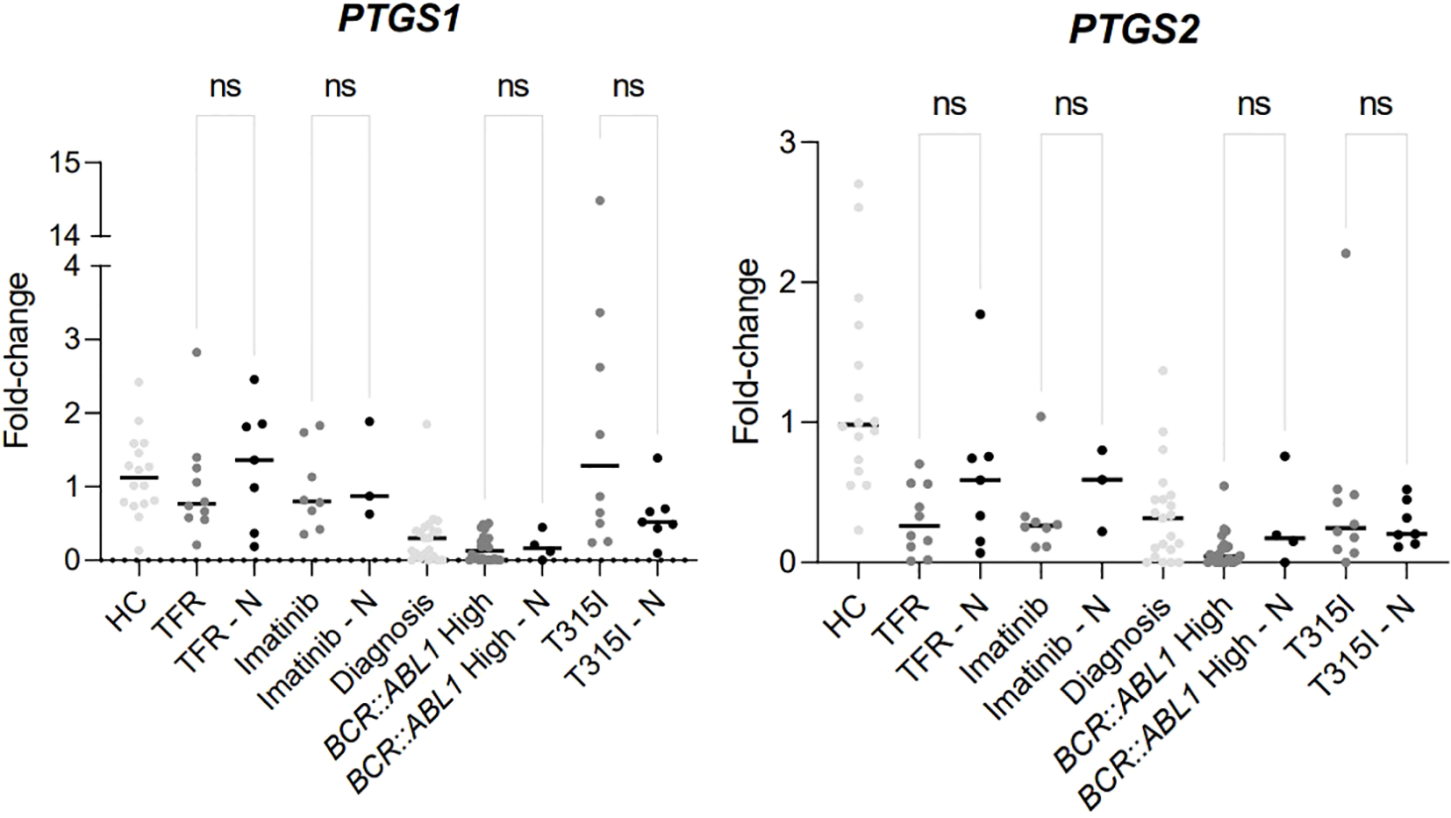
Difference of expression between the analytical groups comparing patients using NSAIDs or not. HC/controls, healthy blood donors; TFR, patients who achieved a good and functional response to imatinib and have begun a treatment-free remission protocol; TFR – N, patients who achieved a good and functional response to imatinib and have begun a treatment-free remission protocol and were using NSAIDs; imatinib, patients undergoing treatment with imatinib; imatinib – N, patients undergoing treatment with imatinib and were using NSAIDs; diagnosis, patients diagnosed with CML but not treated; *BCR::ABL1* high, patients with a percentage of *BCR::ABL1* transcript higher than 0.1%; *BCR::ABL1* high – N, patients with a percentage of *BCR::ABL1* transcript higher than 0.1% and were using NSAIDs; T315I, CML patients with the T315I mutation detected. T315I-N, CML patients with the T315I mutation detected and were using NSAIDs. A p-value of <0.05 was considered significant.

The use of nonsteroidal anti-inflammatory drugs (NSAID) was investigated among the groups of CML patients and in healthy individuals. As shown in **Figure 4** (data shown at **Table 5**), there was no significant difference between the NSAID users and non-users within the groups of CML patients, **Table 5** just shown the information about the patients that were using NSAIDs, other patients that are not in the **Table 5** were not using NSAIDs and were not demonstrated.

**Table 5:** Clinical description of comorbidities and medications used by the patients categorized as using NSAIDs.

Group	Comorbidities	Cardiovascular disease	Medications used continuously during the collection period	Anti-inflammatories (NSAIDs)
TFR	SAH	✓	Amlodipine, Hydralazine, Dipyrone and Gabapentin	✓
TFR	Arthritis	X	Leflunomide	✓
TFR	SAH and Arrhythmia	✓	Atenolol, Amitril and Dipyrone	✓
TFR	Arthritis	X	Leflunomide	✓
TFR	Arthritis	X	Leflunomide	✓
TFR	SAH	✓	Amlodipine, Hydralazine and Dipyrone	✓
TFR	SAH	✓	Amlodipine, Hydralazine and Dipyrone	✓
Imatinib	None	X	Acetylsalicylic acid (Aspirin)	✓
Imatinib	Hypothyroidism and DM	X	Euthyrox, Glyphage, Ciprofibrate, Allopurinol and Acetylsalicylic acid (Aspirin)	✓
Imatinib	SAH	✓	Atenolol, Amlodipine and Dipyrone	✓
*BCR::ABL1* High	HIV and Hypothyroidism	X	Antiretroviral therapy (ART) and Levoid	✓
*BCR::ABL1* High	None	X	Acetylsalicylic acid (Aspirin)	✓
*BCR::ABL1* High	DM and CVA	✓	Metformin, Acetylsalicylic acid (Aspirin), Atorva and Insulin	✓
*BCR::ABL1* High	Hypothyroidism and DM	X	Euthyrox, Glyphage, Ciprofibrate, Allopurinol and Acetylsalicylic acid (Aspirin)	✓
T315I	SAH, AMI, Glaucoma, COPD and DM	✓	Bisoprolol, Acetylsalicylic acid (Aspirin), Atorvastatin, Enalapril, Monocordil, Vastarel and Metformin	✓
T315I	SAH, DM and FP	✓	Nesina, Amlopidino, Metoprolol, Losartan, Gabapentin, Dipyrone, Rivaroxaban, and Simvastatin	✓
T315I	None	✓	Sedated Covid 19 ICU patient	✓
T315I	SAH, DM, Arrhythmia, Dyslipidemia and HF	✓	Pantoprazole, Levothyroxine, Fluoxetine, Metformin, Cilostazol, Eliquis, Losartan, Simvastatin and Bisoprolol	✓
T315I	None	X	Diclofenac	✓
T315I	None	X	Diclofenac	✓
T315I	None	X	Diclofenac	✓

TFR, patients who achieved a good and functional response to imatinib and have begun a treatment-free remission protocol; imatinib, patients undergoing treatment with imatinib; *BCR::ABL1* high, patients with a percentage of *BCR::ABL1* transcript higher than 0.1%; T315I, CML patients with the T315I mutation detected; SAH, Systemic arterial hypertension; DM, Diabetes Mellitus; CVA, Cerebrovascular Accident; AMI, Acute Myocardial Infarction; COPD, Chronic Obstructive Pulmonary Disease; FP, Facial Paralysis; HF, Heart Failure.

### 3.3 Imanitib *in vitro* evaluation

To compose the evidence framework regarding the possible relationship between the expression of *PTGS1* and *PTGS2* genes and CML disease status, an *in vitro* study was performed using the K562 cell model to evaluate the expression of both genes according to the time of exposure to TKI (Imatinib mesylate). For that, first we determined the IC50 for Imatinib mesylate in K562 cells, which was 0.4µM.

**Figure 5:**
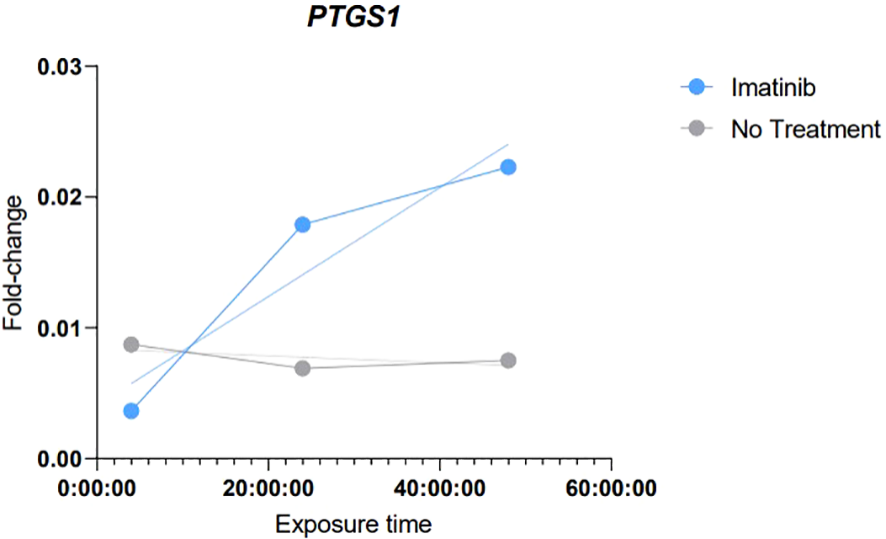
Graphical demonstration of the results of simple linear regression demonstrating the correlation trend of those obtained in the analyses of *in vitro* exposure to Imatinib. Imatinib, Samples with exposure to IC50 of Imatinib. No Treatment, Control samples without any added treatment.

No expression of *PTGS2* was found in the K562 cell line, despite the uniform and high expression levels of the endogenous control gene evaluated (Cycle threshold = 27.8 ± 1.1), which indicates the high and stable RNA quality of the samples. On the other hand, *PTGS1* was expressed in the same cells, as demonstrated in **Tables 6**, **7** and **Figure 5**.

**Table 6:** Numerical demonstration of the Fold-Change results obtained in the *in vitro* exposure analyses to Imatinib (Calculated using the 2^-ΔCT^ methodology).

Sample	Time of exposure	*PTGS1* Expression (2^-ΔCT^)
Imatinib	4h	0,003646
Imatinib	24h	0,017885
Imatinib	48h	0,022294
No Treatment	4h	0,008729
No Treatment	24h	0,006903
No Treatment	48h	0,0075

Imatinib, Samples with exposure to IC50 of Imatinib. No Treatment, Control samples without any added treatment.

**Table 7:** Numerical demonstration of the Pearson correlation results showing the R and R Square values obtained in the *in vitro* exposure analyses to Imatinib.

Group	R	R Square
Imatinib	0,9401	0,8838
No Treatment	-0,6197	0,3841

Imatinib, Samples with exposure to IC50 of Imatinib. No Treatment, Control samples without any added treatment.

The results obtained in these analyses demonstrated a tendency towards an increase of *PTGS1* expression levels, which gradually increased with the longer exposure time to Imatinib. Strong evidence of correlation (R: 0.9401 and R square: 0.8838) and a continuous linear regression trend was observed according to the exposure time.

## 4 Discussion

It is well established that cancers can arise in inflammatory environments and that the tumor microenvironment also promotes inflammation to support tumor cell survival (5). In this context, cyclooxygenase enzymes COX-1 and COX-2 play key roles in the arachidonic acid metabolic pathway and contribute to inflammation through the synthesis of pro-inflammatory molecules such as prostaglandins and thromboxanes (10–12). The genes *PTGS1*, located on chromosome 9, and *PTGS2*, on chromosome 1, encode COX-1 and COX-2, respectively, enabling distinct regulation of these enzyme isoforms (13). Cyclooxygenases (COX) facilitate the conversion of arachidonic acid into prostaglandins, with COX-1 constitutively expressed in normal tissues, while COX-2 is primarily associated with inflammatory and neoplastic processes (14) *PTGS1* and *PTGS2* encode for the respective enzymes COX-1 and COX-2 (15).

This study aimed to evaluate the expression of *PTGS1* and *PTGS2* in clinical samples from CML patients. While COX-1 is constitutively expressed, as it performs housekeeping functions that are essential for homeostasis and to maintain the basal levels of prostanoids in normal cells, COX-2 is predominantly responsive to inflammatory stimuli induced by signals from cytokines, growth factors and tumor-derived molecules (7, 10, 13, 16).

The results for *PTGS1* expression suggest its potential as a biomarker in CML. Specifically, this gene was upregulated in groups of patients with controlled *BCR::ABL1* levels (imatinib, TFR) and in healthy controls, compared to the groups of patients with higher *BCR::ABL1* levels (*BCR::ABL1* high and diagnosis groups). This finding indicates a possible link between inflammatory modulation and CML progression, supporting the hypothesis that patients experiencing a favorable disease progression have an increased *PTGS1* expression.

Although the T315I group did not show significant differences in *PTGS1* expression compared to the *BCR::ABL1* controlled groups, it exhibited an upregulation when compared to the *BCR::ABL1* high ()? group. This observation indicates that the T315I mutation might also influence or be influenced by the inflammation in CML, irrespective of *BCR::ABL1* levels.

Lower *PTGS2* expression was found in patients with uncontrolled *BCR::ABL1* levels (T315I, *BCR::ABL1* high, and diagnosis groups) compared to HCs. Additionally, the *BCR::ABL1* high group showed significant downregulation of *PTGS2* compared to the three *BCR::ABL1* controlled groups (imatinib, TFR, and HC). These findings suggest that *PTGS1* and *PTGS2* work in a similar or coordinated manner in response to CML treatment or progression. The use of NSAIDs, however, was not significantly different within the CML groups.

As a complementary analysis, seeking additional evidence for the observed relationship between *PTGS1* and *PTGS2* expression in CML clinical samples, *in vitro* tests were performed to evaluate the expression of both genes in the K562 cell line when exposed to Imatinib. These results demonstrated that there is a trend and positive correlation between the exposure time to Imatinib and the expression levels of *PTGS1*, i.e,. the longer the time of exposure to the drug, the higher were *PTGS1* levels, this result can be interpreted as complementary evidence to the findings found in clinical samples, considering that these results showed increased *PTGS1* expression in the patients with a good response to TKI treatment. In this case, the increase in the time of exposure to Imatinib would resemble the good response to treatment in the cell line, in which *PTGS1* tends to increase. *PTGS2* was not found expressed at any time in the cell line.

Cyclooxygenases play a central role in inflammatory processes, and the link between cancer and inflammation (16) has prompted investigations about *PTGS1* and *PTGS2* expression across many cancer types (16). A search in the Gene Expression Profile Interactive Analysis (GEPIA, http://gepia.cancer-pku.cn/index.html) database revealed that *PTGS1* is upregulated in ovary and breast cancers but downregulated in bladder cancer, colon adenocarcinoma, and sarcoma. Saindane and colleagues conducted a multi-omic approach to assess the comprehensive median expression of *PTGS2* across 30 cancer types. They found that the expression fluctuates across cancers, with some types exhibiting upregulation and others downregulation (6). Specifically, GEPIA showed *PTGS2* downregulation in bladder, breast, colon, lung, and prostate cancers and upregulation in oesophageal carcinoma, stomach adenocarcinoma, and head and neck squamous cell carcinoma. Although GEPIA indicated *PTGS2* upregulation in acute myeloid leukaemia (AML), no data was available for CML.

Regarding the connection with cancer development, COX-derived eicosanoids can either promote or inhibit carcinogenesis, depending on the receptors they activate. For instance, thromboxane and prostaglandin F tend to have pro-carcinogenic effects, while prostacyclin and prostaglandins D and E are associated with anti-carcinogenic effects (11). Concurrently, arachidonic acid has been shown to inhibit cell growth and induce apoptosis in cell lines that contain the *BCR::ABL1* translocation, which is the main characteristic of CML (17). In solid tumors, COX-2 has been observed to modulate cell proliferation and apoptosis, with higher COX-2 levels leading to increased prostaglandin production and reduced free arachidonic acid (13, 17, 18). Studies on these types of tumors indicate that COX-2 overexpression may predict more advanced stages of the disease and poorer prognosis (7, 12).

To our knowledge, the only report using biological samples from CML patients indicated that *PTGS1* gene expression can differentiate between imatinib-responsive and primary imatinib-resistant cases (19). *PTGS1* has previously been shown to be transcriptionally upregulated by *BCR::ABL1 in vitro* (20). Ramon and colleagues reviewed the expression of PTGS2 and its blockade using non-steroidal anti-inflammatory drugs (NSAIDs) in haematological cancers, highlighting their pro-apoptotic, anti-proliferative, and anti-carcinogenic properties (7). Ross and colleagues assessed the association of NSAID use with the risk of leukaemia, reporting that aspirin was associated with a decreased risk of AML in women but not in men, with a similar pattern observed for CML. No significant associations were found with ibuprofen or COX-2 inhibitors for either sex or leukaemia subtype. The use of these drugs was assessed based on a self-administered questionnaire regarding the type and frequency of NSAID use, along with other demographic questions. The occurrence of leukaemia was determined using a surveillance system. Despite the small number of cases, the authors identified an increased risk of CML associated with the use of COX-2 inhibitors in women. They discussed the disparities between their findings and those of other studies in the literature, which vary based on the type of NSAID used and the reduced or increased risk of leukaemia, and suggest that COX-1 inhibition is most relevant (21). Accordingly, data from the Vitamins and Lifestyle (VITAL) study, which included 64,839 subjects, indicated that high usage (≥ 4 days/week for ≥ 4 years) of acetaminophen increased the risk of haematological malignancies, including myeloid neoplasms and mature B-cell neoplasms, but not chronic lymphocytic leukaemia/small lymphocytic lymphoma (22).

In our data, the use of NSAID did not show significant differences in PTGS genes expression in the different groups of patients. Despite the limited number of samples, this finding may indicate that PTGS expression variations found among the CML patients are mostly influenced by leukemia itself and by the use of Imatinib.

The antiproliferative and pro-apoptotic effects of specific COX-2 inhibitors in cancer cells from solid tumours have been investigated (7, 13, 23), as *PTGS2* gene is overexpressed in breast carcinoma, melanoma, pancreatic, prostate, colon and lung cancer (11, 13, 16, 18). Upregulation of COX-2 is associated with defense against apoptosis and increased cell proliferation, which indicates its influence on oncogenesis (11, 24). It is known that COX-2 plays a relevant part in the progression of lung carcinoma and colon and breast cancers, and low *PTGS2* expression is used as a prognostic marker in the latter (6). The enzyme COX-2, which promotes tumor growth, invasion and metastasis, is highly expressed in triple-negative breast cancer; however, the mechanisms responsible for this increased expression are unknown (6).

However, data regarding haematological malignancies are limited (13). Studies on cancers of haematological origin have recently demonstrated elevated COX-2 expression compared to normal cells (18). In chronic lymphocytic leukaemia (CLL), the overexpression of COX-2 is associated with the increased survival of leukemic B cells (18). Analyses of mRNA using RT-qPCR and microarray, along with Western Blot and immunohistochemistry, confirmed the increased expression of COX-2 in B lymphocytes purified from the peripheral blood of CLL patients (24). Similarly, a correlation between COX-2 expression, tumour proliferation and survival was identified in Hodgkin’s lymphoma. Conversely, in non-Hodgkin’s lymphoma, COX-2 expression was correlated with a worse response to chemotherapy but not with overall survival or tumour grade (18).

There are several reports on the *in vitro* effects of NSAIDs and the expression of *PTGS1* and *PTGS2* in leukaemia cell lines. Focusing on CML, one report showed that K-562 cells induced to imatinib resistance (IR-K562) showed overexpression of COX-2 and *PTGS2*. These IR-K-562 cells were more sensitive to celecoxib alone or in combination with imatinib than the original K-562, imatinib sensitive cells, suggesting that *PTGS2* induction is related to resistance to imatinib (25). This suggests. Subhashini and colleagues also used K-562 cells to demonstrate the antileukemic effects of celecoxib by cell cycle arrest, caspase-3 activation, and downregulation of COX-2 expression (26). Celecoxib has been shown to inhibit colony formation of tyrosine-kinase-resistant cell lines, including those harboring the T315I *BCR::ABL1* mutation. Importantly, celecoxib suppressed colony formation of progenitor hematopoietic cells from CML patients while sparing most CD34+ progenitors from healthy donors (23). Considering these reports, it is reasonable to hypothesize that *PTGS2* downregulation may be useful to evaluate imatinib response and investigate mechanisms of resistance in CML.

The variable evidence in the literature shows that the action of the cyclooxygenases has not yet been fully elucidated, and insufficient evidence exists to clarify their pro- or anti-tumour mechanisms, which are undoubtedly specific to each cancer type. Nonetheless, there are several indications of a relationship between these enzymes and various cancer types. This study provides evidence for the differential expression of *PTGS1* and *PTGS2* in CML in patients with distinct levels of response to treatment. Consequently, this research may pave the way for future studies involving different cohorts and larger sample numbers.

## 5 Conclusion

This work evaluated various CML cohorts and cell line tests, demonstrating significant differences in *PTGS1* and *PTGS2* gene expression. Our results indicate a possible relationship between *PTGS1* and imatinib treatment, as well as between *PTGS2* and the pathophysiology of CML. Future studies are necessary to explore the mechanisms involved in the regulation of both genes over the course of CML.

## Data Availability

The original contributions presented in the study are included in the article/supplementary material, further inquiries can be directed to the corresponding author/s.
